# Medical Devices Applying for Outpatient Medicare Supplemental Payments

**DOI:** 10.1001/jamahealthforum.2024.4016

**Published:** 2024-11-15

**Authors:** Osman Moneer, James L. Johnston, Vinay K. Rathi, Joseph S. Ross, Sanket S. Dhruva

**Affiliations:** 1 Department of Medicine, Stanford University, Stanford, California; 2 Department of Medicine, Brigham and Women’s Hospital, Boston, Massachusetts; 3 Department of Otolaryngology-Head and Neck Surgery, The Ohio State University Wexner Medical Center, Columbus; 4 Section of General Medicine, Department of Medicine, Yale School of Medicine, New Haven, Connecticut; 5 Yale National Clinician Scholars Program, Yale School of Medicine, New Haven, Connecticut; 6 Center for Outcomes Research and Evaluation, Yale−New Haven Hospital, New Haven, Connecticut; 7 Department of Health Policy and Management, Yale School of Public Health, New Haven, Connecticut; 8 School of Medicine, University of California, San Francisco; 9 Section of Cardiology, Department of Medicine, San Francisco Veterans Affairs Health Care System, San Francisco, California

## Abstract

**Importance:**

Medicare transitional pass-through payments (TPTPs) provide supplemental reimbursement that is intended to facilitate adoption of new devices in the outpatient setting. The US Centers for Medicare & Medicaid Services (CMS) have historically evaluated manufacturer applications for TPTPs based on newness, cost, and evidence of substantial clinical improvement, ie, the traditional pathway. In 2020, CMS introduced an alternative pathway to allow US Food and Drug Administration (FDA)-designated breakthrough devices to qualify for supplemental reimbursement without demonstrating substantial clinical improvement.

**Objective:**

To characterize CMS TPTP approval rates and the premarket evidence used by FDA to support authorization of new outpatient medical devices considered for CMS TPTP.

**Design and Setting:**

This was a cross-sectional study of TPTP applications for new outpatient medical devices from 2017 to 2023. Using the Federal Register, CMS Outpatient Prospective Payment System final rules for fiscal years 2017 through 2023 were obtained, from which all manufacturer applications for TPTPs were identified. For each application, the CMS TPTP review pathway (traditional/alternative), CMS final decision (award/deny), and FDA authorization pathway were assessed.

**Main Outcomes and Measures:**

Characteristics of devices considered for CMS TPTPs and design, effectiveness end points, and patient demographic characteristics of premarket clinical studies used to support FDA authorization.

**Results:**

CMS approved 17 of 43 (40%) applications for TPTPs, including all 8 (100%) alternative pathway applications for breakthrough devices and 9 of 35 applications (26%) using the traditional pathway. Devices approved for TPTPs were more likely to have been assessed in premarket clinical studies than devices denied TPTPs (12/17 [71%] vs 2/26 [8%]). Among the 14 premarket studies of TPTP-approved devices, 8 (57%) used surrogate markers as primary effectiveness end points and 5 (42%) did not meet all primary end points. The median (IQR) percentage of female, Black, and Hispanic patients among the trials that reported demographic data was 26% (17%-36%), 6% (2%-17%), and 4% (3%-5%), respectively.

**Conclusions and Relevance:**

The findings of this cross-sectional analysis indicated that CMS more commonly awarded supplemental outpatient payment through TPTPs for devices assessed in premarket clinical studies to support FDA authorization. However, these studies often lacked generalizability to Medicare beneficiaries, used surrogate markers of effectiveness, or did not meet all primary end points. As more breakthrough devices receive FDA authorization and effectively qualify for automatic supplemental payments, strengthening premarket clinical evidence requirements for CMS TPTP approvals would provide better information to guide clinical decision-making and ensure that supplemental reimbursement enhances care for Medicare beneficiaries.

## Key Points

**Question:**

What evidence do the Centers for Medicare & Medicaid Services (CMS) consider when approving or denying transitional pass-through payments (TPTPs) for new outpatient medical devices?

**Findings:**

This cross-sectional study of 43 TPTP applications from 2017 to 2023 found that CMS approved 17 (40%), including all 8 (100%) applications for US Food and Drug Administration (FDA)-designated breakthrough devices. TPTP-approved devices were more likely to have received FDA authorization based on premarket clinical studies, although these studies often lacked generalizability to Medicare beneficiaries, used surrogate markers of effectiveness, or did not meet all primary end points.

**Meaning:**

These findings suggest that strengthening premarket clinical evidence requirements for CMS TPTP approvals would provide better information to guide clinical decision-making and ensure that supplemental device reimbursement enhances care for Medicare beneficiaries.

## Teaser

This cross-sectional study characterizes Medicare transitional pass-through payment approval rates and the premarket evidence used by US Food and Drug Administration to support authorization of new outpatient medical devices.

## Introduction

By statute, the US Centers for Medicare & Medicaid Services (CMS) covers Food and Drug Administration (FDA)−authorized medical devices that it considers reasonable and necessary to care for Medicare beneficiaries.^1^ CMS provides reimbursement for devices through payments that bundle device costs into aggregate amounts that include the professional or facility fees associated with the accompanying procedures.^2^ Because CMS sets reimbursement amounts based on historical costs, the agency sometimes provides supplemental payments for newly marketed devices, which are often more costly than standard care, until it can update existing rates or create new reimbursement codes. The CMS transitional pass-through payments (TPTPs) program^3,4^ provides supplemental payments for novel devices used in outpatient care.

Until 2020, CMS reviewed all TPTP applications for devices using the following required criteria (eBox and eTable 1 in Supplement 1): use (eg, implantation/insertion in a patient), newness (ie, FDA authorized within ≤3 years), cost, and demonstration of “substantial clinical improvement” for Medicare beneficiaries.^3^ After 2020, this traditional pathway was modified to promote device innovation and access through an “alternative” pathway^3^ that allows devices designated under FDA’s Breakthrough Devices Program (BDP) to bypass the substantial clinical improvement requirement.^5^ Under the BDP, FDA expedites device development through multiple features (eg, priority review) and accepts “a greater extent of uncertainty of the benefit-risk profile” based on “adequate postmarket controls to support premarket approval.”^6^

TPTP applications were nonpublic until 2016, so little is known about devices considered for outpatient supplemental payments. Therefore, we examined the presence and design of premarket clinical studies that supported FDA authorization and postmarket study experience with devices applying for TPTPs, comparing those approved and denied.

## Methods

This cross-sectional study used descriptive statistics to summarize data. This research used publicly available data that are nonidentifiable and not human subject research (45 CFR 46.102), and therefore, was exempt from institutional review board approval. This study adhered to the Strengthening the Reporting of Observational Studies in Epidemiology (STROBE) reporting guideline for cross-sectional studies.

### Data Sources and Study Sample

Using the Federal Register, we obtained CMS Outpatient Prospective Payment System final rules for fiscal years 2017 through 2023, from which we identified all manufacturer applications for TPTPs. We abstracted the following information for each application: CMS’ TPTP review pathway (traditional/alternative), CMS’ final decision (award/deny), and FDA authorization pathway.

### Pivotal Clinical Trial and Postmarket Data

We assessed the premarket clinical evidence supporting each device that qualified for TPTP. Using the Devices@FDA database^7^ and peer-reviewed publications, we abstracted the following pivotal study characteristics: randomization, blinding, control(s), sites, and longest duration of primary effectiveness end point follow-up. We categorized each primary effectiveness end point by components (composite/noncomposite), type (surrogate marker/clinical outcome), and result (met/not met).^8^ We abstracted the total number of patients and key demographic characteristics, which were collected from the FDA database and peer-reviewed publications. We assessed whether there was an upper age exclusion limit and/or subgroup analysis by age because more than 85% of CMS’ beneficiary population is 65 years or older.^9^ For each device, we used FDA databases to determine whether FDA required manufacturers to conduct any postmarketing studies (eTable 1 in Supplement 1)^10,11^ and assessed their status as of September 2024.

## Results

### TPTP Application and Medical Device Characteristics

Between 2017 and 2023, manufacturers of 40 devices submitted 43 applications for TPTPs (eFigure in Supplement 1). CMS awarded TPTPs for 17 (40%) of 43 applications, including 9 (26%) of 35 under the Traditional Pathway and 8 (100%) of 8 under the Alternative Pathway for FDA-authorized Breakthrough devices (eTables 2 and 3 in Supplement 1). Of 26 applications denied, 19 (73%) were rejected for not demonstrating substantial clinical improvement in Medicare beneficiaries.

Of 40 unique devices considered by CMS for TPTPs, 12 (30%) received FDA authorization through the premarket approval (PMA) pathway for high-risk devices and 20 (50%) through the 510(k) process for moderate-risk devices (Table 1). Devices authorized under PMA were more likely to be awarded TPTPs compared to 510(k) devices (10/12 [83%] vs 6/20 [30%]).

**Table 1.** Characteristics of Devices for Which Manufacturers Applied for Medicare Transitional Pass-Through Payments

Characteristic	No. (%)		
	All devices (N = 40)	CMS-approved TPTPs (n = 17)	CMS-denied TPTPs (n = 23)
Timing of TPTP application, median (IQR), mo			
FDA authorization to CMS receipt of application	21 (4-32)	4 (1-19)	30 (21-34)
FDA authorization to CMS decision	30 (17-40)	20 (11-31)	37 (26-41)
CMS receipt of application to CMS decision	11 (8-16)	11 (8-17)	11 (8-15)
Corresponding NTAP application			
Did not apply for NTAP	27 (68)	6 (35)	21 (91)
Applied and denied NTAP	1 (3)	0	1 (4)
Applied and approved NTAP	12 (30)	11 (65)	1 (4)
Classified as life sustaining	3 (8)	2 (12)	1 (4)
Therapeutic area			
Dermatological	8 (20)	1 (6)	7 (30)
Cardiovascular	7 (18)	5 (29)	2 (9)
Musculoskeletal	7 (18)	3 (18)	4 (17)
Gastrointestinal	5 (13)	2 (12)	3 (13)
Oncologic	4 (10)	1 (6)	3 (13)
Neurological	3 (8)	2 (12)	1 (4)
Genitourinary	2 (5)	2 (12)	0
Ear nose & throat	2 (5)	0 (0)	2 (9)
Ophthalmologic	1 (3)	1 (6)	0
Psychiatric	1 (3)	0	1 (4)
FDA marketing authorization pathway			
Premarket approval	12 (30)	10 (59)	2 (9)
De novo	3 (8)	1 (6)	2 (9)
510(k)	20 (50)	6 (35)	14 (61)
HCT/Ps	4 (10)	0	4 (17)
Class I exempt	1 (3)	0	1 (4)
Breakthrough designation^a^	8 (47)	8 (47)	0
FDA authorization supported by a pivotal clinical trial	14 (35)	12 (71)	2 (9)
Device subject to FDA postmarketing studies	9 (23)	7 (41)	2 (9)

Abbreviations: CMS, Centers for Medicare & Medicaid Services; FDA, Food and Drug Administration; HCT/Ps, human cells, tissues, and cellular and tissue-based products; NTAP, new technology add-on payment; TPTP, transitional pass-through payments.

^a^ All breakthrough devices in this sample were approved by CMS for TPTPs using the alternative pathway.

### Premarket Clinical Trials

Devices that CMS approved for TPTPs more often had been assessed in premarket clinical studies to support FDA authorization than devices denied TPTPs (12/17 [71%] vs 2/23 [9%]) (Table 1). Among the 17 TPTP-approved devices, 5 devices (29%), all of which were FDA-authorized through the 510(k) process, were not evaluated by pivotal trials.

### Pivotal Clinical Trials for Approved TPTPs

#### Trial Designs

Nearly all (13/14 [93%]) pivotal trials supporting the 12 devices CMS approved for TPTPs were randomized and 5 (36%) were blinded (Table 2). The median (IQR) duration of primary effectiveness end point follow-up was 6 (3-6) months; 8 (57%) used any surrogate markers for primary effectiveness end points, 5 (36%) of which solely used surrogate markers.

**Table 2.** Trial Design Characteristics of Pivotal Trials for Devices Approved for CMS TPTPs

Characteristic	All pivotal trials, No. (%)	Pivotal trial for approved TPTP, by pathway, No. (%)	
		Traditional	Alternative
Pivotal trials, No.	14	6	8
Randomized	13 (93)	6 (100)	7 (88)
Controlled	13 (93)	6 (100)	7 (88)
Active	5 (36)	3 (50)	2 (25)
Sham	4 (28)	3 (50)	1 (13)
Placebo	4 (28)	0	4 (50)
Blinding			
Double	3 (21)	2 (33)	0
Single	2 (14)	1 (17)	2 (25)
None	9 (64)	3 (50)	6 (75)
Multicenter	14 (100)	6 (100)	8 (100)
Use of surrogate markers for all primary effectiveness end points	5 (36)	2 (33)	3 (38)
Composite end point	6 (43)	4 (67)	2 (25)
Duration of follow-up, median (IQR), mo	6 (3-6)	6 (6-6)	5 (3-6)
Inferential pivotal trials	12	6	6
Trial comparator design			
Equivalence	0	0	0
Noninferiority	4 (33)	3 (50)	1 (17)
Superiority	8 (67)	3 (50)	5 (83)
Trial met all primary effectiveness end points	7 (58)	4 (67)	3 (50)
Trial met any primary effectiveness end point	9 (75)	4 (67)	5 (63)
Primary effectiveness end points	18	6	12
Clinical outcome	10 (55)	4 (67)	6 (50)
Surrogate marker	8 (44)	2 (33)	6 (50)

Abbreviations: CMS, Centers for Medicare & Medicaid Services; TPTP, transitional pass-through payments.

#### Treatment Effectiveness

Treatment effect estimates compared to concurrent controls were reported in 12 pivotal trials for 10 TPTP-approved devices, 7 (58%) of which met all and 9 (75%) of which met any primary effectiveness end point (eTable 4 in Supplement 1). Among the 5 trials that did not meet all primary effectiveness end points, 2 (40%) failed criteria for noninferiority and 3 (60%) for superiority.

#### Demographic Characteristics

Among the 14 pivotal trials supporting FDA authorization of TPTP-approved devices, the median (IQR) number of patients enrolled in the investigational arm was 109 (69-258) (Table 3). The median (IQR) age of trial populations was 60 (56-64) years; no pivotal trials included subgroup analyses by age 65 years or older, and 3 excluded patients (21%) were 80 years or older. Among trials that reported demographic characteristics, the median (IQR) percentage of participants was as follows: female, 26% (17%-36%); Black or African American, 6% (2%-17%), and Hispanic or Latino, 4% (3%-5%).

**Table 3.** Demographic Characteristics of 14 Pivotal Trials for 12 Devices Approved for CMS TPTPs

Characteristic	All pivotal trials, No. (%)	Pivotal trial for approved TPTP, by pathway, No. (%)	
		Traditional	Alternative
Pivotal trials, No.	14	6	8
Total patients, median (IQR)	171 (138-413)	167 (151-356)	272 (106-413)
Participants in investigational arm, median (IQR)	109 (69-258)	95 (69-233)	116 (69-258)
Excluded patients on dialysis	3 (21)	1 (17)	2 (25)
**Age, y**			
Age data reported	14 (100)	6 (100)	8 (100)
Age of trial population, median (IQR)	60 (56-64)	62 (57-66)	59 (46-61)
Upper age exclusion limit	3 (21)	2 (33)	1 (13)
Upper age exclusion limit, median (IQR)	80 (80-80)	80 (80-80)	80 (80-80)
Any subgroup analyses by age	2 (14)	0	2 (25)
Subgroup analyses by age ≥65	0	0	0
**Sex, median (IQR), %**			
Sex data reported	14 (100)	6 (100)	8 (100)
Female	26 (17-36)	40 (16-49)	24 (18-30)
**Race, median (IQR), %**			
Race data reported	11 (79)	3 (50)	8 (100)
American Indian or Alaska Native	1.2 (0.5-2.0)	2.2 (2.2-2.2)	0.5 (0.5-1.2)
Asian	2.9 (0.02-3.4)	0	3.2 (2.2-3.5)
Black or African American	6.0 (2.4-17.4)	6 (6.0-6.0)	9.9 (2.1-17.8)
Native Hawaiian or Pacific Islander	0.2 (0.1-0.2)	0	0.3 (0.2-0.3)
Other^a^	1.9 (1.5-8.4)	1.5 (1.5-1.5)	4.4 (1.7-9.2)
White	77.8 (68.0-87.6)	91 (88.6-93)	70.9 (64.6-79.1)
**Ethnicity, median (IQR), %**			
Ethnicity data reported	4 (29)	1 (17)	3 (38)
Hispanic or Latino	4.1 (3.2-4.8)	6.7 (6.7-6.7)	4.0 (2.3-4.1)

Abbreviations: CMS, Centers for Medicare & Medicaid Services; TPTP, transitional pass-through payments.

^a^ Other was the term used in the US Food and Drug Administration and other data sources, and its definition may have varied between sources; this study did not define the term.

### Postmarket Study Experience

Devices approved for TPTPs were more often subject to FDA-required postmarketing studies compared to those denied TPTPs (7/17 [41%] vs 2/23 [9%]). Of the 10 postmarketing studies for TPTP-approved devices, 5 (50%) were completed, 2 (20%) ongoing, 2 (20%) delayed, and 1 (10%) terminated as of September 2024; the median (IQR) time to completion was 36 (22-55) months.

## Discussion

In this cross-sectional study of the entire sample of medical devices considered by CMS for supplemental reimbursement through the TPTP program from 2017 to 2023, more than one-fourth of TPTP-approved devices were not assessed in pivotal clinical studies, despite all being surgically inserted or implanted. All of these devices were authorized via the 510(k) process and some were exempted from demonstrating “substantial clinical improvement” due to their FDA breakthrough designation. Although many TPTP-approved devices were evaluated in pivotal studies, critical questions often remained unanswered because these studies had short follow-up, often used surrogate markers of effectiveness, did not meet all primary end points, and/or enrolled younger patients that were not representative of Medicare beneficiaries.

CMS could consider several strategies to ensure that supplemental payments are reserved for technologies that meaningfully enhance the care of Medicare beneficiaries. First, the agency could establish minimum evidence standards for TPTP devices, requiring a clinical outcome benefit in at least 1 premarket clinical study that enrolls patients who are representative of Medicare beneficiaries and that uses an active comparator. Second, CMS could consider case-by-case rather than universal exemptions from the substantial clinical improvement requirement for breakthrough devices. Third, CMS could associate the annual TPTP renewal process to adequate postmarket study progress,^12^ thereby incentivizing manufacturers to generate much-needed clinical evidence in a timely manner.^13^

### Limitations

This study was limited by reliance on publicly available documents. Although CMS may have considered additional data, this information was not consistently reported in federal rules. Nevertheless, because sponsors apply for TPTPs shortly (median 4 months for TPTP-approved devices) after FDA authorization, we do not anticipate that additional evidence was routinely available to CMS.

## Conclusions

The findings of this cross-sectional study suggest that as more breakthrough devices receive FDA authorization^14^ and effectively qualify for automatic supplemental payments, CMS should consider strengthening premarket evidence requirements to help inform treatment decisions and improve patient care. Not only would stronger evidence requirements provide better information to guide clinical decision-making, they would also ensure that supplemental device reimbursement improves care for Medicare beneficiaries.

## Notes

Supplement 1. eMethods eBox. Eligibility Criteria for Transitional Pass-through Payments eFigure. Applications for Medicare Transitional Pass-Through Payments by Year and CMS Approval Pathway Over Time eTable 1. Key Terms for FDA Regulation and Medicare Coverage of Medical Devices eTable 2. Devices Approved and Denied for CMS TPTPs, 2017 to 2023 eTable 3. Characteristics of Devices Approved for TPTPs through Traditional vs Alternative Pathways (N=17) eTable 4. Devices Approved for CMS TPTPs With Pivotal Trials Not Meeting All Primary Effectiveness Endpoints (N= 5) eReferences

Supplement 2. Data Sharing Statement
