# Supplementary material for: Medical Devices Applying for Outpatient Medicare Supplemental Payments
Source: JAMA Health Forum. 2024 Nov 15;5(11):e244016. doi: 10.1001/jamahealthforum.2024.4016 (PMC11568453; doi:10.1001/jamahealthforum.2024.4016)
Supplement: Supplement 1. — eMethods eBox. Eligibility Criteria for Transitional Pass-through Payments eFigure. Applications for Medicare Transitional Pass-Through Payments by Year and CMS Approval Pathway Over Time eTable 1. Key Terms for FDA Regulation and Medicare Coverage of Medical Devices eTable 2. Devices Approved and Denied for CMS TPTPs, 2017 to 2023 eTable 3. Characteristics of Devices Approved for TPTPs through Traditional vs Alternative Pathways (N=17) eTable 4. Devices Approved for CMS TPTPs With Pivotal Trials Not Meeting All Primary Effectiveness Endpoints (N= 5) eReferences [file jamahealthforum-e244016-s001.pdf]

Supplemental Online Content

Moneer O, Johnston JL, Rathi VK, Ross JS, Dhruva SS. Medical Devices Applying for Outpatient Medicare Supplemental Payments. *JAMA Health Forum.* Published online November 15, 2024. doi:10.1001/jamahealthforum.2024.4016

eMethods

eBox. Eligibility Criteria for Transitional Pass-through Payments

eFigure. Applications for Medicare Transitional Pass-Through Payments by Year and CMS Approval Pathway Over Time

eTable 1. Key Terms for FDA Regulation and Medicare Coverage of Medical Devices

eTable 2. Devices Approved and Denied for CMS TPTPs, 2017 to 2023

eTable 3. Characteristics of Devices Approved for TPTPs through Traditional vs Alternative Pathways (N=17)

eTable 4. Devices Approved for CMS TPTPs With Pivotal Trials Not Meeting All Primary Effectiveness Endpoints (N= 5)

eReferences

This supplemental material has been provided by the authors to give readers additional information about their work.

© 2024 Moneer O et al. *JAMA Health Forum*.

Table of Contents

eMethods......3

eBox: Eligibility Criteria for Transitional Pass-through Payments......5

eFigure: Applications for Medicare Transitional Pass-Through Payments by Year and CMS Approval Pathway Over Time......6

eTable 1: Key Terms for FDA Regulation and Medicare Coverage of Medical Devices......7

eTable 2: Devices Approved and Denied for CMS TPTPs, 2017 to 2023......8

eTable 3: Characteristics of Devices Approved for TPTPs through Traditional vs Alternative Pathways (N=17)......12

eTable 4: Devices Approved for CMS TPTPs With Pivotal Trials Not Meeting All Primary Effectiveness Endpoints (N= 5)......14

eReferences......16

© 2024 Moneer O et al. *JAMA Health Forum*.

eMethods

Transitional Pass-Through Payment (TPTP) Application and Medical Device Characteristics

We abstracted the product name, sponsor, and corresponding therapeutic area using the World Health Organization Anatomical Therapeutic Chemical Classification scheme.[1] We also abstracted the duration of time from Food and Drug Administration (FDA) authorization to Centers for Medicare and Medicaid Services (CMS) receipt and/or approval of TPTP application.

Premarket Data Abstraction

For each medical device, we retrieved the product classification codes and marketing authorization decision summary from the Devices@FDA database[2] and extracted the characteristics of all premarket pivotal clinical trials, which generally serve as the primary basis for FDA authorization. If specific data elements were unavailable in FDA decision summaries, we used the published literature. We identified trial publications by searching PubMed for product names (e.g., AngelMed Guardian®) and/or trial names (e.g., BeAT-HF) and validated by comparing to information in FDA decision summaries.

For each pivotal trial, we evaluated if outcomes were composites or surrogates. Composite measures combine multiple outcomes (e.g., myocardial infarction, stroke, or death) into a single endpoint.[3] Clinical outcome measures assess how patients feel, function, or survive, while surrogate markers measure effects on a biomarker or imaging test (e.g., tumor size) intended to predict a clinical outcome. If a trial had more than one primary effectiveness endpoint, we noted whether any of the individual endpoints were surrogate markers.

© 2024 Moneer O et al. *JAMA Health Forum*.

For each pivotal trial that included a concurrent control arm and therefore permitted inferences of effectiveness, we abstracted the comparator type (active/sham/placebo) and whether the trial utilized a non-inferiority, equivalence, or superiority design. We also determined whether the treatment effect estimates for any primary effectiveness endpoints were statistically significant, noting if any or all endpoints were met.

Among characteristics of pivotal trial participants, we also determined whether patients on dialysis were excluded, because end-stage renal disease requiring dialysis or kidney transplant is a criterion for Medicare eligibility.[4]

Postmarket Data Abstraction

We used FDA databases to determine whether FDA required manufacturers to conduct any postmarketing studies (through either post-approval studies to address safety and/or effectiveness questions for high-risk devices or Section 522 postmarket surveillance studies to address safety questions for moderate- or high-risk devices).[5,6] We identified these studies and their FDA-designated completion status (complete/ongoing/delayed/terminated) as of September 2024.

© 2024 Moneer O et al. *JAMA Health Forum*.

eBox: Eligibility Criteria for Transitional Pass-through Payments

42 CFR 419.66 Transitional pass-through payments: Medical devices

(b) *Eligibility.* A medical device must meet the following requirements:

(1) If required by the FDA, the device must have received FDA premarket approval or clearance (except for a device that has received an FDA investigational device exemption (IDE) and has been classified as a Category B device by the FDA in accordance with §§ 405.203 through 405.207 and 405.211 through 405.215 of this chapter), or meet another appropriate FDA exemption for premarket approval or clearance. Under this provision, the pass-through payment application for a medical device must be submitted within 3 years from the date of the initial FDA approval or clearance, if required, unless there is a documented, verifiable delay in U.S. market availability after FDA approval or clearance is granted, in which case CMS will consider the pass-through payment application if it is submitted within 3 years from the date of market availability.

(2) The device is determined to be reasonable and necessary for the diagnosis or treatment of an illness or injury or to improve the functioning of a malformed body part (as required by section 1862(a)(1)(A) of the Act).

(3) The device is an integral part of the service furnished, is used for one patient only, comes in contact with human tissue, and is surgically implanted or inserted (either permanently or temporarily) or applied in or on a wound or other skin lesion.

(4) The device is not any of the following:

(i) Equipment, an instrument, apparatus, implement, or item of this type for which depreciation and financing expenses are recovered as depreciable assets as defined in Chapter 1 of the Medicare Provider Reimbursement Manual (CMS Pub. 15–1).

(ii) A material or supply furnished incident to a service (for example, a suture, customized surgical kit, or clip, other than radiological site marker).

© 2024 Moneer O et al. *JAMA Health Forum*.

eFigure: Applications for Medicare Transitional Pass-Through Payments by Year and CMS Approval Pathway Over Time

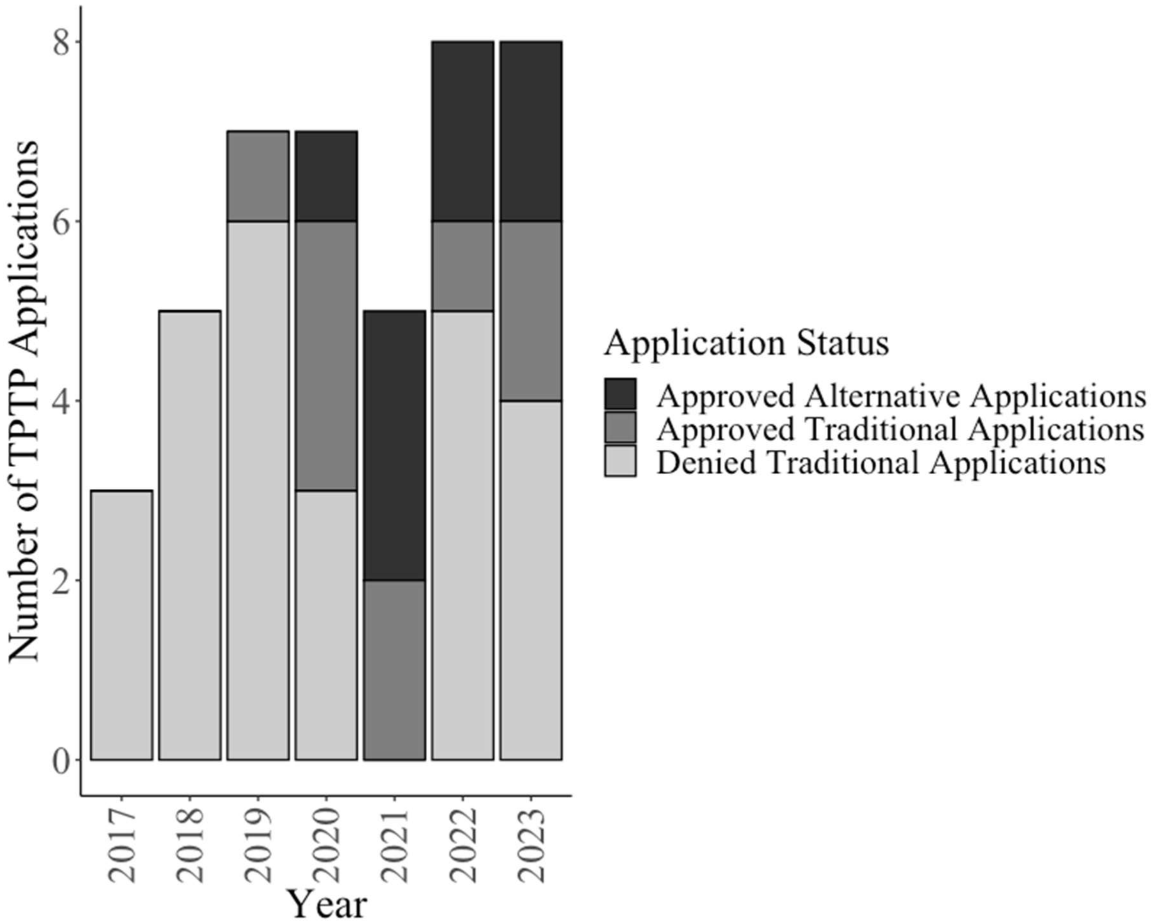

Abbreviations: TPTP, Transitional Pass-through Payments; CMS, Centers for Medicare and Medicaid Services

© 2024 Moneer O et al. *JAMA Health Forum*.

eTable 1: Key Terms for Food and Drug Administration (FDA) Regulation and Medicare Coverage of Medical Devices

Term	Description
**Premarket regulation**	
Class I Exempt	Most low-risk devices are not subject to FDA premarket review.
De Novo	Novel low and moderate-risk devices typically must be studied in clinical trials prior to receiving market authorization.[7]
510(k)	Low and moderate-risk devices may obtain market authorization by demonstrating "substantial equivalence" to predicate devices that have previously been legally marketed. [8]
Premarket Approval	High-risk devices must prove reasonable assurance of safety and effectiveness. [9]
**Postmarket regulation**	
Section 522 Postmarket Surveillance Studies	FDA may require postmarket surveillance requirements for moderate or high-risk devices that are (1) "likely to have serious adverse health consequences" upon failure, (2) used in "pediatric populations," (3) "intended to be implanted in the body for more than a year," or (4) are "intended to be life-sustaining or life-supporting used outside a device user facility." [10]
Post-Approval Studies	FDA may require manufacturers of high-risk devices subject to Premarket Approval to conduct post-approval studies.
**Medicare Payment**	
Transitional Pass-Through Payments (TPTPs) & New Technology Add-on Payments (NTAP)	CMS may award supplemental payments to devices that are (1) new, (2) "not insignificant" in cost, and (3) "demonstrate substantial clinical improvement." Devices authorized by FDA after designation in the Breakthrough Devices Program are exempt from meeting the third criterion, for substantial clinical improvement.[11] CMS issues TPTPs to devices used in the outpatient setting and NTAPs to devices used in the inpatient setting. [12]

© 2024 Moneer O et al. *JAMA Health Forum*.

eTable 2: Devices Approved and Denied for CMS Transitional Pass-Through Payments, 2017 to 2023

Device Name	Sponsor	Year Reviewed for TPTPs	Pathway
	TPTP-Approved Devices		
Remedē® System Transvenous Neurostimulator	Respicardia, Inc.	2019	Traditional
Surefire® Spark™ Infusion System	TriSalus Life Sciences	2020	Traditional
Optimizer® System	Impulse Dynamics	2020	Alternative
AquaBeam® System	PROCEPT BioRobotics Corporation	2020	Traditional
AUGMENT® Bone Graft	Wright Medical	2020	Traditional
CUSTOM FLEX® ARTIFICIAL IRIS	VEO Ophthalmics	2021	Alternative
EXALT™ Model D Single-Use Duodenoscope	Boston Scientific Corporation	2021	Alternative
BAROSTIM NEO™ System	CVRx, Inc.	2021	Alternative
Hemospray® Endoscopic Hemostat	Cook Medical	2021	Traditional
SpineJack® Expansion Kit	Stryker, Inc	2021	Traditional
RECELL® System	AVITA Medical	2022	Alternative
Shockwave C2 Coronary Intravascular	Shockwave Medical	2022	Alternative

© 2024 Moneer O et al. *JAMA Health Forum*.

Lithotripsy (IVL) Catheter			
AngelMed Guardian® System	Angel Medical Systems	2022	Traditional
aprevo™ Intervertebral Body Fusion Device	Carlsmed, Inc	2023	Alternative
MicroTransponder® ViviStim® Paired Vagus Nerve Stimulation (VNS) System (Vivistim® System)	MicroTransponder, Inc.	2023	Alternative
Evoke® Spinal Cord Stimulation (SCS) System	Saluda Medical Inc	2023	Traditional
The Uretero1	STERIS	2023	Traditional
	TPTP-Denied Devices		
Endophys Pressure Sensing System	Endophys Holdings, LLC	2017	Traditional
BioBag® (Larval Debridement Therapy in a Contained Dressing)	BioMonde US, LLC	2017	Traditional
Encore™ Suspension System	Siesta Medical, Inc	2017	Traditional
Architect® Px	Harbor MedTech, Inc	2018	Traditional
Dermavest and Plurivest Human Placental Connective Tissue Matrix (HPCTM)	Aedicell, Inc	2018	Traditional
Kerecis® Omega3 Wound (Skin Substitute)	Kerecis, LLC	2018	Traditional

© 2024 Moneer O et al. *JAMA Health Forum*.

FlōGraft®/Flōgraft Neogenesis®	Applied Biologics, LLC	2018	Traditional
X-WRAP®	Applied Biologics, LLC	2018	Traditional
EpiCord®	MiMedx®	2019	Traditional
BlastX ™ Antimicrobial Wound Gel	Next ScienceTM	2019	Traditional
Restrata® Wound Matrix	Acera Surgical, Inc	2019	Traditional
SpaceOAR® System	Augmenix, Inc	2019	Traditional
AquaBeam System	PROCEPT BioRobotics Corporation	2019	Traditional
BioBag® (Larval Debridement Therapy in a Contained Dressing)	BioMonde US, LLC	2019	Traditional
Eluvia™ Drug-Eluting Vascular Stent System	Boston Scientific Corporation	2020	Traditional
TracPatch	TracPatch Health	2020	Traditional
Vagus Nerve Stimulation (VNS) Therapy® System for Treatment Resistant Depression (TRD)	LivaNova USA Inc	2020	Traditional
BONEBRIDGE Bone Conduction Implant System	MED-EL Corporation	2022	Traditional
Cochlear™ Osia® 2 System	Cochlear Americas	2022	Traditional
Pure-Vu® System	Motus GI	2022	Traditional
Xenoscope™	Xenocor Inc.	2022	Traditional

© 2024 Moneer O et al. *JAMA Health Forum*.

Eluvia™ Drug-Eluting Vascular Stent System	Boston Scientific Corporation	2022	Traditional
Pathfinder® Endoscope Overtube	Neptune Medical, Inc	2023	Traditional
The BrainScope TBI (Model: Ahead 500)	BrainScope Company Inc.	2023	Traditional
NavSlim™ and NavPencil	Elucent Medical, Inc	2023	Traditional
SmartClip™	Elucent Medical, Inc	2023	Traditional

© 2024 Moneer O et al. *JAMA Health Forum*.

eTable 3: Characteristics of Devices Approved for TPTPs through Traditional vs Alternative Pathways (N=17)

Characteristic	All Approved Devices N = 17	Traditional Pathway, N = 9	Alternative Pathway, N = 8
	N (%) or Median (IQR)		
**Timing of TPTP Application**			
Time from FDA authorization to CMS receipt of TPTP application (months)	4 (1 – 19)	9 (3 – 21)	2 (0 – 8)
Time from FDA authorization to CMS decision for TPTP application (months)	20 (11 – 31)	23 (13 – 31)	15 (10 – 26)
**Classified as Life-Sustaining**	2 (12)	0 (0)	2 (25)
**Therapeutic Area**			
Dermatological	1 (6)	0 (0)	1 (13)
Cardiovascular	5 (29)	2 (22)	3 (38)
Musculoskeletal	3 (18)	2 (22)	1 (13)
Gastrointestinal	2 (12)	1 (11)	1 (13)
Oncologic	1 (6)	1 (11)	0 (0)
Neurological	2 (12)	1 (11)	1 (13)
Genitourinary	2 (12)	2 (22)	0 (0)
Ear Nose & Throat	0 (0)	0 (0)	0 (0)
Ophthalmologic	1 (6)	0 (0)	1 (13)
Psychiatric	0 (0)	0 (0)	0 (0)
**FDA Marketing Authorization Pathway**			
Premarket Approval	10 (59)	4 (44)	6 (75)
De Novo	1 (6)	1 (11)	0 (0)
510(k)	6 (35)	4 (44)	2 (25)
HCT/Ps	0 (0)	0 (0)	0 (0)

© 2024 Moneer O et al. *JAMA Health Forum*.

Class I Exempt	0 (0)	0 (0)	0 (0)
**FDA Authorization Supported by a Pivotal Clinical Trial**	12 (71)	6 (67)	6 (75)
Premarket Approval	10 of 10 (100)	4 of 4 (100)	6 of 6 (100)
De Novo	1 of 1 (100)	1 of 1 (100)	0 of 0 (NA)
510(k)	1 of 6 (17)	1 of 4 (25)	0 of 2 (0)
**Device Subject to FDA Postmarketing Studies**	7 (41)	3 (33)	4 (50)

Abbreviations: TPTP, Transitional Pass-through Payments; FDA, Food and Drug Administration; CMS, Centers for Medicare and Medicaid Services; NTAP, New Technology Add-On Payment; HCT/Ps, Human cells, tissues, and cellular and tissue-based products.

© 2024 Moneer O et al. *JAMA Health Forum*.

eTable 4: Devices Approved for CMS TPTPs With Pivotal Trials Not Meeting All Primary Effectiveness Endpoints (N= 5)

**Device name**<br><br>**[TPTP application pathway, TPTP approval year]**	**FDA-approved indications for use**	**Description of unmet primary effectiveness endpoint**
Optimizer® Smart System<br><br>[Alternative, 2020]	"To improve 6-minute hall walk distance, quality of life, and functional status of NYHA Class III heart failure patients who remain symptomatic despite guideline directed medical therapy, who are in normal sinus rhythm, are not indicated for Cardiac Resynchronization Therapy, and have a left ventricular ejection fraction ranging from 25% to 45%."	Change from baseline in the ventilatory anaerobic threshold (VAT) measured on CPX
AUGMENT® Bone Graft<br><br>[Traditional, 2020]	"For use as an alternative to autograft in arthrodesis (i.e., surgical fusion procedures) of the ankle (tibiotalar joint) and/or hindfoot (including subtalar, talonavicular, and calcaneocuboid joints, alone or in combination), due to osteoarthritis, post-traumatic arthritis, rheumatoid arthritis, psoriatic arthritis, avascular necrosis, joint instability, joint deformity, congenital defect, or joint arthropathy in patients with preoperative or intraoperative evidence indicating the need for supplemental graft material."	Proportion of patients with fusion and a non-inferiority margin of 10%. Fusion was assessed using CT imaging for the full complement of joints, defined as evaluating all joints, and was classified as a success only if all treated joints possessed at least 50% bridging osseous bone
Barostim Neo® System<br><br>[Alternative, 2021]	"For the improvement of symptoms of heart failure – quality of life, six-minute hall walk and functional status, for patients who remain symptomatic despite treatment with guideline-directed medical therapy, are NYHA Class III or Class II (who had a recent history of Class III), have a left ventricular ejection fraction ≤35%, a NT-proBNP < 1600 pg/ml and excluding patients indicated for Cardiac Resynchronization Therapy (CRT) according to AHA/ACC/ESC guidelines."	Difference in change in NT-pro-BNP between Barostim plus medical management group vs medical management only group

© 2024 Moneer O et al. *JAMA Health Forum*.

AngelMed Guardian® System<br><br>[Traditional, 2022]	"For use in patients who have had prior acute coronary syndrome (ACS) events and who remain at high risk for recurrent ACS events.<br>The Guardian System is indicated as an adjunct to patient recognized symptoms. The Guardian System detects potential ongoing ACS events, characterized by sustained ST segment changes, and alerts the patient to seek medical attention for those potential ACS events."	Composite endpoint of cardiac/unexplained death, new Q-wave myocardial infarction, or detection to presentation time >2 hours
RECELL® System<br><br>[Alternative, 2022]	"For the treatment of acute thermal burn wounds in patients 18 years of age and older. The RECELL® Device is used by an appropriately-licensed healthcare professional at the patient's point-of-care to prepare autologous Regenerative Epidermal Suspension (RES™) for direct application to acute partial-thickness thermal burn wounds or application in combination with meshed autografting for acute full-thickness thermal burn wounds."	Non-inferiority of RECELL® relative to control for recipient site healing using a pre-specified non-inferiority margin of -10%

Abbreviations: NYHA, New York Heart Association; CPX, Cardiopulmonary Exercise Testing; VAT, Ventilatory Anaerobic Threshold; CT, Computed Tomography; NT-proBNP, N-terminal pro B-type Natriuretic Peptide; AHA, American Heart Association; ACC, American College of Cardiology; ESC, European Society of Cardiology; CRT, Cardiac Resynchronization Therapy; ACS, Acute Coronary Syndrome; RES, Regenerative Epidermal Suspension.

© 2024 Moneer O et al. *JAMA Health Forum*.

eReferences

1. WHO Collaborating Centre for Drug Statistics Methodology. ATC/DDD Index. Accessed June 7, 2024. https://www.whocc.no/atc_ddd_index/

2. US Food and Drug Administration. Devices@FDA. Accessed June 7, 2024. https://www.accessdata.fda.gov/scripts/cdrh/devicesatfda/index.cfm

3. US Food and Drug Administration. Multiple Endpoints in Clinical Trials. April 15, 2024. Accessed June 7, 2024. https://www.fda.gov/regulatory-information/search-fda-guidance-documents/multiple-endpoints-clinical-trials

4. US Centers for Medicare & Medicaid Services. Who's eligible for Medicare? February 9, 12AD. Accessed June 7, 2024. https://www.hhs.gov/answers/medicare-and-medicaid/who-is-eligible-for-medicare/index.html

5. Post-Approval Studies (PAS) Database. Accessed June 7, 2024. https://www.accessdata.fda.gov/scripts/cdrh/cfdocs/cfPMA/pma_pas.cfm

6. US Food and Drug Administration. 522 Postmarket Surveillance Studies Database. Accessed June 7, 2024. https://www.accessdata.fda.gov/scripts/cdrh/cfdocs/cfPMA/pss.cfm

7. US Food and Drug Administration. De Novo Classification Request. FDA. October 3, 2022. Accessed June 7, 2024. https://www.fda.gov/medical-devices/premarket-submissions-selecting-and-preparing-correct-submission/de-novo-classification-request

8. US Food and Drug Administration. Premarket Notification 510(k). FDA. December 5, 2023. Accessed June 7, 2024. https://www.fda.gov/medical-devices/premarket-submissions-selecting-and-preparing-correct-submission/premarket-notification-510k

9. US Food and Drug Administration. Premarket Approval (PMA). FDA. January 5, 2023. Accessed June 7, 2024. https://www.fda.gov/medical-devices/premarket-submissions-selecting-and-preparing-correct-submission/premarket-approval-pma

10. US Food and Drug Administration. 522 Postmarket Surveillance Studies Program. FDA. October 4, 2022. Accessed June 7, 2024. https://www.fda.gov/medical-devices/postmarket-requirements-devices/522-postmarket-surveillance-studies-program

11. *42 CFR § 419.66 - Transitional Pass-through Payments: Medical Devices.*

12. *42 CFR § 412.88. Additional Payment for New Medical Service or Technology.*

© 2024 Moneer O et al. *JAMA Health Forum*.
