# Supplementary material for: Medical Devices Applying for Outpatient Medicare Supplemental Payments
Source: JAMA Health Forum. 2024 Nov 15;5(11):e244016. doi: 10.1001/jamahealthforum.2024.4016 (PMC11568453; doi:10.1001/jamahealthforum.2024.4016)
Supplement: Supplement 2. — Data Sharing Statement [file jamahealthforum-e244016-s002.pdf]

Data Sharing Statement

Moneer. Medical Devices Applying for Outpatient Medicare Supplemental Payments. *JAMA Health Forum*. Published November 15, 2024. doi:10.1001/jamahealthforum.2024.4016

Data
Data available: No

Additional Information
Explanation for why data not available: We utilized publicly available sources for this study, including the Federal Register, Devices@FDA database, and published literature. We will make the data underlying the publication available upon publication, and requests should be directed to the corresponding author.
